# Tinnitus and Cognition in the Elderly: Unraveling the Impact of Symptom Burden on Cognitive Decline

**DOI:** 10.3390/brainsci15080869

**Published:** 2025-08-15

**Authors:** Sarah Alshehri, Abdulaziz Abdulrahman S. Al Hatem

**Affiliations:** Otolaryngology, Head and Neck Surgery, Department of Surgery, College of Medicine, King Khalid University, Abha 61421, Saudi Arabia; 442809848@kku.edu.sa

**Keywords:** tinnitus, cognitive impairment, aging, Mini-Cog, THI, hearing loss

## Abstract

Background/Objectives: Tinnitus has been increasingly recognized not only as an auditory disturbance but also as a condition that is potentially linked to cognitive decline, particularly in older adults. However, the relationship between tinnitus characteristics and cognitive impairment remains underexplored in clinical settings. This study aimed to (1) evaluate the association between chronic tinnitus and cognitive function in adults aged 60 years and above using standardized tools, and (2) determine whether tinnitus severity and duration are associated with increased risk of cognitive impairment. Methods: A cross-sectional study was conducted among 240 older adults, divided into tinnitus (*n* = 120) and non-tinnitus (*n* = 120) groups. Cognitive function was assessed using the Mini-Cog (0–5) and SPMSQ (0–10) tools. Tinnitus severity and duration were evaluated using the Tinnitus Handicap Inventory (THI), Visual Analogue Scale (VAS), and duration categories. Results: Participants with tinnitus were significantly older and had higher rates of hearing loss (58.33% vs. 33.33%, *p* = 0.001), depression (37.50% vs. 18.33%, *p* = 0.002), and poor sleep quality (51.67% vs. 31.67%, *p* = 0.003). Mini-Cog and SPMSQ scores were significantly lower in the tinnitus group (2.87 ± 1.14 vs. 3.52 ± 1.06; 6.95 ± 1.42 vs. 8.02 ± 1.18; both *p* < 0.001). Tinnitus presence, longer duration, and higher severity were independently associated with cognitive impairment. Each 10-point increase in THI score increased the odds of impairment by 45% (OR = 1.45, *p* < 0.001). Conclusions: Tinnitus burden, particularly when severe and prolonged, is significantly associated with cognitive impairment in older adults. These findings highlight the need for cognitive screening and integrated management in this population.

## 1. Introduction

Tinnitus, characterized by the perception of sound in the absence of an external auditory stimulus, affects an estimated 10–15% of the global adult population, with prevalence rates rising to 30% or higher among older adults [1]. While often regarded primarily as a benign auditory symptom, chronic tinnitus is increasingly recognized for its substantial impact on quality of life, encompassing sleep disturbance, emotional distress, and impaired concentration [2]. In geriatric populations, where comorbidities such as hearing loss, depression, and cardiovascular risk factors are common, tinnitus can represent a multidimensional clinical burden [3]. Cognitive decline in older adults, particularly in domains such as attention, memory, and executive functioning, poses a growing public health concern [4]. As both tinnitus and cognitive impairment are prevalent and debilitating conditions in aging populations, clarifying their potential interrelationship holds significant implications for early detection and holistic management strategies [3].

Emerging literature suggests that chronic tinnitus may be associated with cognitive dysfunction, though the strength and specificity of this relationship remain subjects of ongoing investigation [5]. Neuroimaging studies have revealed that individuals with tinnitus exhibit altered functional connectivity in attention- and memory-related neural networks, including the default mode and salience networks [6]. Li et al. [7] reported disrupted fronto-parietal connectivity in tinnitus patients, which may underlie reduced cognitive efficiency. Behavioral studies further support this connection [8]. Jensen et al. [9] and Brueggemann et al. [10] observed that tinnitus severity correlated with attentional and executive deficits, even after adjusting for hearing loss and age. Similarly, Waechter et al. [11] found that individuals with bothersome tinnitus performed worse on working memory tasks, implicating a cognitive load effect. However, despite such evidence, the independent role of tinnitus characteristics—such as severity, duration, and type—and their interplay with cognitive function remain incompletely defined, particularly in relation to well-validated cognitive screening tools used in clinical settings [12].

Importantly, prior research has often failed to adequately account for confounding variables such as hearing impairment, depression, and sleep disturbance—factors that are both prevalent among tinnitus sufferers and independently linked to cognitive decline [13,14]. Furthermore, few studies have employed rigorous regression modeling to isolate the unique contribution of tinnitus to cognitive function while adjusting for relevant demographic and clinical covariates [15,16]. This limitation restricts the clinical applicability of existing findings [16]. There is a critical need for studies that not only quantify the cognitive performance of older adults with tinnitus using standardized instruments but also examine how specific tinnitus-related parameters (duration, severity, laterality, and type) and comorbid conditions affect cognitive outcomes. Such work can inform whether tinnitus may serve as a clinical marker for cognitive screening and guide the development of integrated intervention strategies.

The present study addresses this gap by systematically investigating the association between tinnitus and cognitive function in adults aged 60 years and older. Using the Mini-Cog and SPMSQ—two brief, validated cognitive screening tools—this study aims to (1) assess differences in cognitive function between individuals with and without chronic tinnitus, and (2) evaluate whether tinnitus severity and duration are associated with increased risk of cognitive impairment, independent of age, hearing loss, depression, and other clinical covariates. We hypothesized that participants with tinnitus, especially those with more severe or prolonged symptoms, would demonstrate lower cognitive scores and higher rates of cognitive impairment compared to their non-tinnitus counterparts.

## 2. Materials and Methods

### 2.1. Study Design, Ethics, and Settings

This cross-sectional study was conducted between August 2024 and May 2025 at the KKU medical city clinics of Otolaryngology, King Khalid University, Kingdom of Saudi Arabia. Ethical approval for the study was obtained from the Institutional Review Board of King Khalid University (IRB approval number: [ECM#2025-712]), and written informed consent was obtained from all participants prior to data collection. The study adhered strictly to the ethical principles outlined in the Declaration of Helsinki, ensuring respect for participant autonomy, confidentiality, and the right to withdraw without penalty.

### 2.2. Sample Size

The sample size for this cross-sectional study was determined using G*Power version 3.1.9.7 software (Heinrich Heine University, Düsseldorf, Germany), based on a two-tailed independent samples *t*-test for detecting differences in cognitive function scores (Mini-Cog and SPMSQ) between participants with and without tinnitus [15]. Assuming a moderate effect size of 0.40, which was derived from prior literature indicating clinically meaningful differences in cognitive outcomes among tinnitus populations, a significance level (α) of 0.05, and a statistical power (1 − β) of 0.90, the required sample size was calculated to be 108 participants per group. To account for potential exclusions due to incomplete data or protocol deviations, a 10% buffer was added, resulting in a final target sample size of 120 individuals in the tinnitus group and 120 in the non-tinnitus group, totaling 240 participants.

### 2.3. Participants

Participants for this study were recruited through consecutive sampling from the outpatient otolaryngology and geriatric medicine clinics at King Khalid University Hospital between June 2023 and April 2024. Eligible individuals were approached during routine consultations and invited to participate following an initial review of their clinical history and presenting symptoms. Participants with tinnitus were identified based on a clinical diagnosis confirmed by an otolaryngologist, supported by self-reported persistent perception of sound without an external source lasting for at least six months. Tinnitus severity and duration were further assessed using the Tinnitus Handicap Inventory (THI) [17] and a Visual Analogue Scale (VAS), which are validated instruments for quantifying symptom impact and intensity [18].

Inclusion criteria were as follows: age 60 years or older, ability to provide informed consent, and sufficient auditory and cognitive ability to complete study assessments. For the tinnitus group, participants were required to have a diagnosis of subjective or objective tinnitus, with symptom duration clearly categorized (<6 months, 6–12 months, 1–5 years, or >5 years). The non-tinnitus control group consisted of age- and sex-matched individuals attending the same clinic without a current or prior history of tinnitus. A structured screening interview was conducted with control participants to confirm the absence of past or current tinnitus symptoms, using standard otologic history and self-report. Exclusion criteria included known neurodegenerative disorders (e.g., Alzheimer’s disease, Parkinson’s disease), acute psychiatric illness, uncorrected hearing loss that precluded reliable communication, or use of ototoxic medications in the past six months.

Prior to enrollment, all participants underwent a structured screening process that included clinical history, otologic examination, and preliminary cognitive assessment to ensure eligibility. Demographic and clinical information, including comorbidities, medication use, and psychosocial variables, was collected at baseline to support detailed analysis and statistical adjustment.

### 2.4. Variables

#### 2.4.1. Primary Outcome Variable

The primary outcome of this study was cognitive function, assessed using two standardized and clinically validated screening tools: the Mini-Cognitive Assessment Instrument (Mini-Cog) [19] and the Short Portable Mental Status Questionnaire (SPMSQ) [20]. The Mini-Cog includes a three-item recall task and a clock-drawing test, with a total score ranging from 0 to 5. One point was awarded for each correctly recalled word and two points for a correctly drawn clock, based on standard scoring criteria [19]. A score of ≤2 was used to indicate potential cognitive impairment, in accordance with established diagnostic cutoffs. The SPMSQ consists of 10 orientation and calculation items, adjusted for education level, with ≥3 errors suggesting possible cognitive impairment [20]. Both tools were administered in a quiet clinical setting by trained assessors, and scores were recorded at the time of enrollment to ensure standardized conditions. All instruments were administered in Arabic, using culturally adapted and previously validated versions to ensure linguistic accuracy.

#### 2.4.2. Secondary Outcome Variables

The secondary outcome was the presence or absence of cognitive impairment, derived from the dichotomization of the Mini-Cog and SPMSQ scores based on validated cutoffs. Participants were classified as cognitively impaired if they scored ≤2 on the Mini-Cog or made ≥3 errors on the SPMSQ, after accounting for educational adjustments. This binary variable served as the dependent variable in logistic regression analyses evaluating risk factors for cognitive decline. Additionally, the prevalence of cognitive impairment across categories of tinnitus severity and duration was analyzed as a categorical outcome.

#### 2.4.3. Independent Variables/Predictors

The key independent variables included tinnitus presence (yes/no), tinnitus severity, tinnitus duration, type (subjective/objective), and laterality (unilateral/bilateral). Tinnitus severity was quantified using the Tinnitus Handicap Inventory (THI) [17], a 25-item instrument producing a total score from 0 to 100, with higher scores reflecting greater perceived burden. Severity was further stratified using Visual Analogue Scale (VAS) ratings from 0 (no tinnitus) to 10 (extreme severity). Tinnitus duration was recorded in months and categorized into four clinically meaningful intervals: <6 months, 6–12 months, 1–5 years, and >5 years. Type and laterality were established through clinical evaluation by an otolaryngologist and patient self-report during structured interviews.

#### 2.4.4. Covariates/Confounders

Several covariates were included to adjust for potential confounding effects in multivariate analyses. These included age (continuous), gender (male/female), education level (no formal, primary, secondary, tertiary), and hearing loss (yes/no), as self-reported and verified through clinical examination. Psychological and health-related variables included depressive symptoms, assessed using the 15-item Geriatric Depression Scale (GDS-15), sleep quality (poor/good) based on subjective report, and presence of comorbidities such as hypertension, diabetes, and stroke. Use of CNS-active medications (e.g., sedatives, antidepressants) and hearing aid use (yes/no) were also included due to their potential influence on cognitive outcomes.

#### 2.4.5. Data Collection Instruments and Procedures

All clinical and cognitive assessments were conducted face-to-face by trained clinicians and researchers in a standardized protocol. Sociodemographic data and medical histories were obtained through structured interviews using pretested data collection forms. Tinnitus-related variables were assessed using validated Arabic-language versions of the THI and VAS, ensuring cultural and linguistic suitability. Cognitive assessments were performed in a quiet, distraction-free environment, with immediate recording of scores to minimize recall bias. Quality assurance checks were implemented throughout data collection to maintain accuracy and consistency across all participants.

### 2.5. Statistical Analysis

Data were analyzed using SPSS version 24, with statistical significance set at *p* < 0.05. For Objective 1, descriptive statistics (mean, standard deviation, frequency, and percentage) were used to summarize the demographic and clinical characteristics of participants. Independent samples t-tests and one-way ANOVA were applied to compare cognitive function scores (Mini-Cog and SPMSQ) based on tinnitus presence, type (subjective or objective), and laterality (unilateral or bilateral). To further explore associations, multiple linear regression was conducted to assess the independent effect of tinnitus presence, type, and duration on cognitive function scores, adjusting for age, gender, education, hearing loss, depression status, and sleep quality. For Objective 2, ANOVA and chi-square tests were used to evaluate the relationship between tinnitus severity and duration (categorized) and the presence of cognitive impairment, defined by validated Mini-Cog and SPMSQ thresholds. Binary logistic regression was then performed to identify predictors of cognitive impairment, using tinnitus severity and duration as main independent variables and adjusting for potential confounders such as hearing aid use, medication, and comorbidities. Categorical variables such as tinnitus laterality, tinnitus type, and comorbidity status were dummy coded prior to inclusion in regression models. Assumptions for multivariate regression, including linearity, independence of errors, and absence of multicollinearity, were verified prior to model interpretation. All continuous variables followed a normal distribution, justifying the use of parametric statistical methods.

## 3. Results

Participants with tinnitus were older than their non-tinnitus counterparts (72.34 ± 6.54 vs. 70.21 ± 5.87 years, d = 0.34, *p* = 0.034) and exhibited markedly higher rates of hearing loss (58.33% vs. 33.33%, V = 0.17, *p* = 0.001) (Table 1). Depression (37.50% vs. 18.33%, V = 0.22, *p* = 0.002) and poor sleep quality (51.67% vs. 31.67%, V = 0.15, *p* = 0.003) were also substantially more prevalent among tinnitus cases. Hypertension occurred in 56.67% of the tinnitus group compared with 43.33% in the non-tinnitus group (V = 0.31, *p* = 0.015), while hearing aid use was reported by 23.33% of tinnitus participants compared to 11.67% of non-tinnitus participants (V = 0.13, *p* = 0.018). No statistically significant differences were observed for gender distribution, educational attainment, diabetes, or stroke history, with effect sizes in these comparisons remaining small.

Participants with tinnitus demonstrated significantly lower cognitive scores compared to those without tinnitus on both the Mini-Cog and SPMSQ assessments (Table 2). Within the tinnitus group, individuals with subjective tinnitus, bilateral involvement, and lower Mini-Cog and SPMSQ scores exhibited greater cognitive impairment relative to those with objective or unilateral tinnitus. These differences were statistically significant, highlighting a potential association between tinnitus characteristics and cognitive performance.

**Figure 1:**
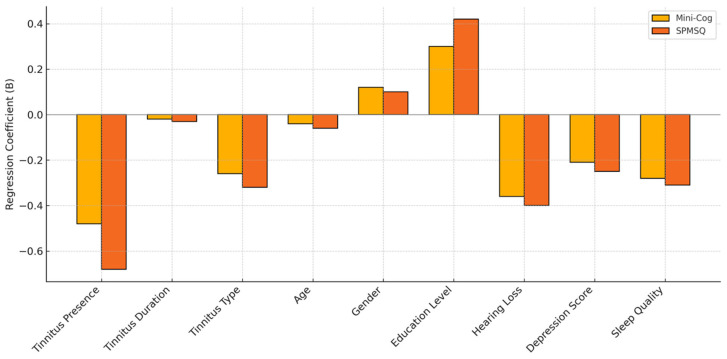
Multivariate linear regression coefficients for predictors of Mini-Cog and SPMSQ Cognitive Scores.

Tinnitus presence, longer tinnitus duration, subjective tinnitus type, older age, hearing loss, depressive symptoms, and poor sleep quality were all independently associated with lower cognitive function scores on both the Mini-Cog and SPMSQ, as indicated by statistically significant negative regression coefficients (Figure 1). Higher education level emerged as a positive predictor of better cognitive performance across both assessments. Gender did not significantly influence cognitive scores. These findings suggest that multiple clinical and demographic variables, particularly those related to tinnitus characteristics and psychosocial factors, may contribute to cognitive decline in older adults.

The prevalence of cognitive impairment increased significantly with both higher tinnitus severity and longer duration, as indicated by progressive elevations in THI scores and corresponding cognitive impairment rates across severity and duration categories (Table 3). Individuals with severe tinnitus reported the highest THI scores and the greatest proportion of cognitive impairment (65.00%), while those with mild tinnitus showed minimal cognitive deficits (13.33%). Similarly, longer tinnitus duration, particularly beyond five years, was associated with markedly higher cognitive impairment prevalence (76.67%), compared to those with symptom duration under six months (10.00%). These findings suggest a strong, graded association between tinnitus burden and cognitive decline.

**Figure 2:**
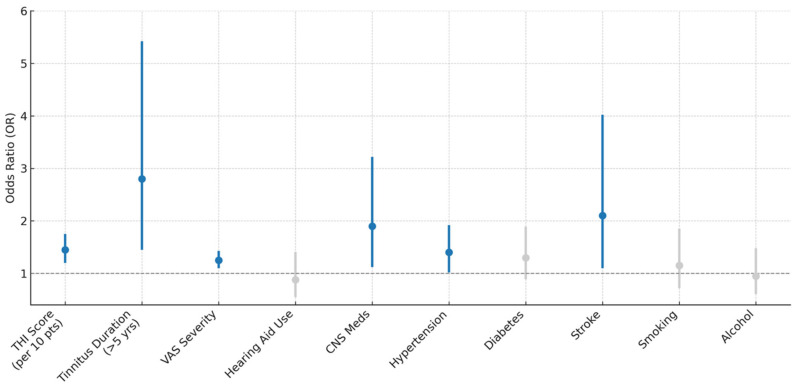
Adjusted odds ratios for cognitive impairment based on tinnitus characteristics and clinical covariates. The colors represent the statistical significance of the predictors: Blue bars: Variables significantly associated with cognitive impairment (*p*-value < 0.05). These predictors have odds ratios (OR) whose 95% confidence intervals do not cross the dashed line at OR = 1. Grey bars: Variables not significantly associated with cognitive impairment (*p*-value ≥ 0.05). Their 95% confidence intervals cross OR = 1, indicating no statistically significant relationship.

Higher tinnitus burden, indicated by increases in THI scores and VAS severity ratings, as well as longer symptom duration (>5 years), was significantly associated with elevated odds of cognitive impairment (Figure 2). Specifically, each 10-point increase in THI score was associated with a 45% increase in the odds of impairment (OR = 1.45, *p* < 0.001), while individuals with tinnitus lasting more than 5 years had nearly threefold higher odds (OR = 2.80, *p* = 0.002). Use of CNS-active medications (OR = 1.90, *p* = 0.017), comorbid hypertension (OR = 1.40, *p* = 0.042), and history of stroke (OR = 2.10, *p* = 0.026) were also significant predictors. In contrast, hearing aid use, diabetes, smoking, and alcohol use were not significantly associated with cognitive impairment.

## 4. Discussion

This cross-sectional study aimed to examine the relationship between chronic tinnitus and cognitive function in older adults, as well as to explore whether the severity and duration of tinnitus symptoms contribute to an elevated risk of cognitive impairment. The findings demonstrated a consistent association between tinnitus and reduced cognitive performance, as measured by validated screening tools. Participants with tinnitus, particularly those with subjective and bilateral presentations, exhibited significantly poorer cognitive scores compared to those without tinnitus. The comparison between subjective and objective tinnitus should be interpreted with caution, as the relatively small number of participants with objective tinnitus (*n* = 20) may limit statistical power to detect differences and increase the likelihood of type II errors. Larger samples are required to more reliably assess subtype-specific associations with cognitive outcomes. Furthermore, regression analyses identified tinnitus presence, longer symptom duration, and higher tinnitus severity as independent predictors of lower cognitive function, even after adjusting for key demographic and clinical covariates. Although age is a well-established contributor to cognitive decline, its effect was statistically adjusted for in all multivariate analyses, allowing the association between tinnitus and cognitive performance to be examined independently of chronological aging. Nevertheless, given the cross-sectional design, the possibility that age-related processes interact with or amplify the cognitive effects of tinnitus cannot be excluded. Additional factors such as advanced age, hearing loss, depressive symptoms, and poor sleep quality also emerged as significant contributors to cognitive decline. In contrast, gender and certain lifestyle factors, including smoking and alcohol use, were not significantly associated with cognitive outcomes. These results highlight the multifactorial nature of cognitive impairment in older adults with tinnitus and underscore the importance of comprehensive assessment in this population.

Participants with tinnitus exhibited significantly poorer cognitive performance, a finding that may be attributed to the complex interplay of neuro-auditory and psychosocial factors associated with chronic tinnitus [5]. Individuals in the tinnitus group were not only older but also had markedly higher rates of hearing loss, depressive symptoms, poor sleep quality, and hypertension—each of which has independently been implicated in cognitive decline [4]. The multivariate analysis confirmed that tinnitus presence, longer symptom duration, and subjective or bilateral tinnitus types were all associated with lower scores on the Mini-Cog and SPMSQ assessments, even after adjusting for confounding variables. Importantly, cognitive performance declined further in individuals with concurrent sensory and psychological burdens, such as hearing loss and depression, suggesting a cumulative effect of tinnitus-related distress and impaired sensory input on cognitive processing [21]. In contrast, gender showed no significant association with cognitive outcomes, while higher educational attainment demonstrated a protective effect, underscoring the moderating role of cognitive reserve.

These findings are consistent with previous research highlighting the association between tinnitus and cognitive dysfunction in older adults. For instance, Jensen et al. [9] reported that chronic tinnitus was linked to lower cognitive performance across attention and memory domains. Similarly, research by Brueggemann et al. [10] emphasized that tinnitus severity correlated with reduced executive functioning, independent of age and hearing loss. A study by Han et al. [22] further confirmed that bilateral and subjective tinnitus were more strongly associated with cognitive deficits, in line with the present study’s subgroup comparisons. Additionally, the observed association between depression and lower cognitive scores aligns with evidence from Cui et al. [23], who demonstrated that depressive symptoms substantially mediate the tinnitus–cognition relationship. The role of poor sleep noted in this study also echoes findings from Li et al. [24], who identified disrupted sleep as a significant contributor to cognitive complaints in patients with tinnitus. Collectively, these parallels strengthen the reliability of the present findings and suggest a need for integrated assessment and management strategies targeting both audiological and cognitive health in this population [25].

The observed increase in cognitive impairment prevalence with higher tinnitus severity and longer duration can be attributed to the cumulative burden imposed by persistent auditory distress on neurocognitive functioning [15]. Severe tinnitus, as reflected by elevated THI and VAS scores, may contribute to chronic cognitive overload, limiting attentional capacity and working memory by continuously diverting neural resources toward intrusive auditory sensations [26]. Prolonged exposure to tinnitus symptoms over several years may further exacerbate cognitive fatigue, promote maladaptive neural plasticity, and disrupt central auditory–cognitive networks [27]. Additionally, the presence of CNS-active medication use, comorbid hypertension, and a history of stroke were significantly associated with greater odds of impairment, suggesting that vascular and pharmacological influences may amplify the neurocognitive impact of tinnitus [28]. These factors likely compound the effects of auditory dysfunction by introducing additional neural stressors that impair executive and memory-related functions [29].

The findings are consistent with prior studies that have demonstrated a dose–response relationship between tinnitus burden and cognitive impairment. A study conducted by Jensen et al. reported that greater tinnitus severity correlated with increased cognitive complaints, particularly in domains of attention and executive function [9]. Xiong et al. [30] used neuroimaging to show that long-term tinnitus is associated with altered connectivity in the default mode and salience networks, which are essential for cognitive regulation [30]. Likewise, Oosterloo et al. [31] found that individuals with severe and long-standing tinnitus exhibited significantly lower Mini-Mental State Examination scores, independent of age and hearing status. The current results regarding comorbidities are in agreement with findings by Del Vecchio et al. [32], who identified vascular risk factors and medication use as significant contributors to cognitive decline in older tinnitus patients. Together, these results reinforce the hypothesis that tinnitus-related distress and comorbid clinical conditions jointly influence the trajectory of cognitive aging.

### 4.1. Clinical Significance

The clinical significance of this study lies in its identification of chronic tinnitus—particularly severe, prolonged, and bilateral forms—as a significant, independent contributor to cognitive impairment in older adults. By demonstrating robust associations between tinnitus burden and lower cognitive scores on both the Mini-Cog and SPMSQ, even after adjusting for key confounders such as age, hearing loss, depression, and sleep quality, the findings highlight the need for routine cognitive screening in elderly patients presenting with tinnitus. Furthermore, the study underscores the importance of early and multidisciplinary management strategies that not only address the perceptual and emotional components of tinnitus but also consider its broader neurocognitive consequences. Recognizing tinnitus as more than an auditory symptom, but rather a potential marker of cognitive vulnerability, can inform clinical decision-making and promote integrated care approaches aimed at preserving cognitive health in geriatric populations.

### 4.2. Limitations

This study offers important insights into the relationship between tinnitus and cognitive function in older adults, but it is subject to several limitations. The cross-sectional design prevents the determination of causality, making it unclear whether tinnitus contributes to cognitive decline or whether cognitive decline influences tinnitus perception. Reliance on self-reported measures for certain variables, including hearing loss, sleep quality, and depression, may introduce reporting bias. Additionally, potentially relevant factors such as social isolation, physical activity, and detailed cardiovascular health profiles were not assessed, precluding sensitivity analyses to evaluate their influence. Unmeasured confounding from such variables may partially account for the observed associations, highlighting the need for future research to include a broader range of social and health-related covariates. Objective neuroimaging and electrophysiological assessments were not incorporated, limiting the mechanistic interpretation of the findings. Generalizability may be restricted by the demographic and clinical homogeneity of the sample, as well as the single-center recruitment in Saudi Arabia, where cultural, environmental, and healthcare system factors—such as variations in occupational and recreational noise exposure, healthcare access, and cultural attitudes toward hearing health—may influence both tinnitus prevalence and its cognitive impact. These contextual considerations should be taken into account when applying the findings to other populations. Longitudinal studies are needed to clarify causal pathways and track the temporal progression of cognitive decline in individuals with tinnitus. Future research using advanced neuroimaging, biomarker analysis, and targeted interventions could further elucidate underlying mechanisms and guide therapy development. While the Mini-Cog and SPMSQ are validated and practical for use in older adults, they provide only a global assessment and do not differentiate between specific cognitive domains such as executive function, memory, or processing speed. Comprehensive neuropsychological batteries in future studies could offer more detailed insights into the cognitive processes most affected. Although potential neurobiological mechanisms, including dysfunction within the default mode and salience networks, are discussed, these were not directly investigated; thus, the mechanistic interpretations remain hypothetical and require empirical evaluation using neuroimaging and electrophysiological methods.

## 5. Conclusions

In conclusion, this study demonstrates that chronic tinnitus in older adults is independently associated with reduced cognitive function, as measured by the Mini-Cog and SPMSQ. The severity, duration, and type of tinnitus, along with factors such as older age, hearing loss, depressive symptoms, and poor sleep quality, were significantly associated with lower cognitive scores and higher odds of cognitive impairment. Higher educational attainment was positively correlated with better cognitive performance, while gender and lifestyle factors such as smoking and alcohol use showed no significant associations. These findings underscore the relevance of tinnitus as a clinical marker for cognitive assessment in the elderly and emphasize the need for comprehensive evaluation in patients presenting with chronic tinnitus.

## Figures and Tables

**Table 1:** Demographic and clinical characteristics of participants by tinnitus status.

Variable	Tinnitus (*n* = 120)	Non-Tinnitus (*n* = 120)	Effect Size	*p*-Value
Age (years)	72.34 ± 6.54	70.21 ± 5.87	0.34	0.034
Gender—Male (%)	64 (53.33)	58 (48.33)	0.29	0.482
Gender—Female (%)	56 (46.67)	62 (51.67)	0.35	0.482
Education—No Formal (%)	8 (6.67)	6 (5.00)	0.33	0.564
Education—Primary (%)	28 (23.33)	24 (20.00)	0.31	0.585
Education—Secondary (%)	52 (43.33)	50 (41.67)	0.18	0.812
Education—Tertiary (%)	32 (26.67)	40 (33.33)	0.2	0.176
Hearing Loss—Yes (%)	70 (58.33)	40 (33.33)	0.17	0.001
Depression—Yes (%)	45 (37.50)	22 (18.33)	0.22	0.002
Poor Sleep Quality—Yes (%)	62 (51.67)	38 (31.67)	0.15	0.003
Hypertension—Yes (%)	68 (56.67)	52 (43.33)	0.31	0.015
Diabetes—Yes (%)	36 (30.00)	30 (25.00)	0.23	0.289
Stroke—Yes (%)	10 (8.33)	6 (5.00)	0.07	0.312
Hearing Aid Use—Yes (%)	28 (23.33)	14 (11.67)	0.13	0.018

SD: standard deviation; *n*: number of participants; %: percentage. Effect sizes are presented as Cohen’s d for continuous variables and Cramér’s V for categorical variables to complement *p*-values and provide an estimate of the magnitude of between-group differences.

**Table 2:** Comparison of cognitive scores by tinnitus presence, type, and laterality.

Group	Mini-Cog Score (Mean ± SD)	SPMSQ Score (Mean ± SD)	*p*-Value (Mini-Cog)	*p*-Value (SPMSQ)
Tinnitus (*n* = 120)	2.87 ± 1.14	6.95 ± 1.42	0.001	0.000
Non-Tinnitus (*n* = 120)	3.52 ± 1.06	8.02 ± 1.18	-	-
Subjective tinnitus (*n* = 100)	2.75 ± 1.12	6.80 ± 1.38	0.038	0.022
Objective Tinnitus (*n* = 20)	3.30 ± 1.10	7.55 ± 1.33	0.038	0.022
Unilateral tinnitus (*n* = 50)	3.10 ± 1.08	7.20 ± 1.35	0.045	0.033
Bilateral tinnitus (*n* = 70)	2.68 ± 1.15	6.75 ± 1.39	0.045	0.033

SD: standard deviation; SPMSQ: Short Portable Mental Status Questionnaire; Mini-Cog: Mini-Cognitive Assessment Instrument; *n*: number of participants; *p*: *p*-value.

**Table 3:** Cognitive impairment prevalence by tinnitus severity and duration.

Tinnitus Category	Mean THI Score ± SD	Cognitive Impairment (%)	*p*-Value (Chi-Square)
Severity (VAS 0–3, Mild; *n* = 30)	18.25 ± 6.80	4 (13.33)	0.001
Severity (VAS 4–6, Moderate; *n* = 50)	42.10 ± 9.75	18 (36.00)	0.001
Severity (VAS 7–10, Severe; *n* = 40)	68.45 ± 11.30	26 (65.00)	0.001
Duration < 6 months (*n* = 20)	22.40 ± 8.10	2 (10.00)	0.001
Duration 6–12 months (*n* = 30)	36.85 ± 10.50	7 (23.33)	0.001
Duration 1–5 years (*n* = 40)	51.30 ± 12.00	16 (40.00)	0.001
Duration > 5 years (*n* = 30)	64.90 ± 11.45	23 (76.67)	0.001

THI: Tinnitus Handicap Inventory; SD: standard deviation; VAS: Visual Analogue Scale; *n*: number of participants; %: percentage; *p*: *p*-value.

## Data Availability

The datasets generated and analyzed during the current study are publicly available in the Zenodo repository. All versions of the dataset can be accessed and cited using the following DOI: [10.5281/zenodo. 16079325] (https://doi.org/10.5281/zenodo.16079325, accessed on 13 August 2025). This ensures transparency and enables full replication of the study findings.

## Abbreviations

The following abbreviations are used in this manuscript:

THI	Tinnitus Handicap Inventory
VAS	Visual Analogue Scale
SPMSQ	Short Portable Mental Status Questionnaire
Mini-Cog	Mini-Cognitive Assessment Instrument
GDS-15	Geriatric Depression Scale-15
CNS	Central Nervous System
